# Editorial: Vegetation-based degradation and restoration on the alpine grasslands of the Tibetan plateau

**DOI:** 10.3389/fpls.2024.1467335

**Published:** 2024-08-01

**Authors:** Yujie Niu, Yanfu Bai, Sergio Rossi

**Affiliations:** ^1^ College of Grassland Science, Key Laboratory of Grassland Ecosystem of the Ministry of Education, Gansu Agricultural University, Lanzhou, Gansu, China; ^2^ Department of Disturbance Ecology, BayCEER, University of Bayreuth, Bayreuth, Germany; ^3^ College of Grassland Science and Technology, Sichuan Agricultural University, Chengdu, China; ^4^ Laboratoire sur les écosystèmes terrestres boréaux, Département des Sciences Fondamentales, Université du Québec à Chicoutimi, Chicoutimi, QC, Canada

**Keywords:** vegetation classification, disturbance, land degradation, climate change, plant-soil interactions

The Tibetan Plateau is the world’s highest and largest plateau, often referred to as ‘the roof of the world’ due to its high elevation and extensive mountain systems, which host hotspots of endemic biodiversity. It is also known as the “third pole” because of its cold environment, and the “Asian water tower” owing to its rich river systems. This unique physical and geographical environment supports a variety of alpine ecosystems, predominantly classified as alpine grasslands (**Figure 1**). These grasslands cover approximately 50-70% of the Tibetan plateau’s total land area (Wu et al., 2021; Zhang et al., 2022). Such a range in estimates arises from varying definitions of grassland according to different authorities and scientists, particularly concerning the inclusion of cropland, desert, or shrublands. Most of these rangelands feature a fragile, tundra-like environment that is difficult to restore once degraded (Niu et al., 2019) due to the slow soil formation processes, low nutrient availability, limited seed recruitment, and the short growing season (Li et al., 2023; Niu et al., 2023a). The main natural grassland types on the Tibetan plateau include alpine deserts, alpine steppes, alpine meadows, alpine swamps, and the transitional types between them varying along the precipitation gradient. The alpine grasslands continue to experience severe degradation due to multiple factors (Liu et al., 2018; Niu et al., 2023b), primarily anthropogenic disturbances (e.g. overgrazing, road construction), as well as natural environmental changes (e.g. climate change, rodent outbreak). As a result, its degradation presents a major global environmental threat given the importance of Tibetan grasslands to biodiversity, ecosystem complexity, and provision of ecosystem services. This Research Topic tried to explore the current state of vegetation-based alpine grassland degradation, the causes of this degradation and possible solutions to restore and re-establish the local plant communities.

**Figure 1:**
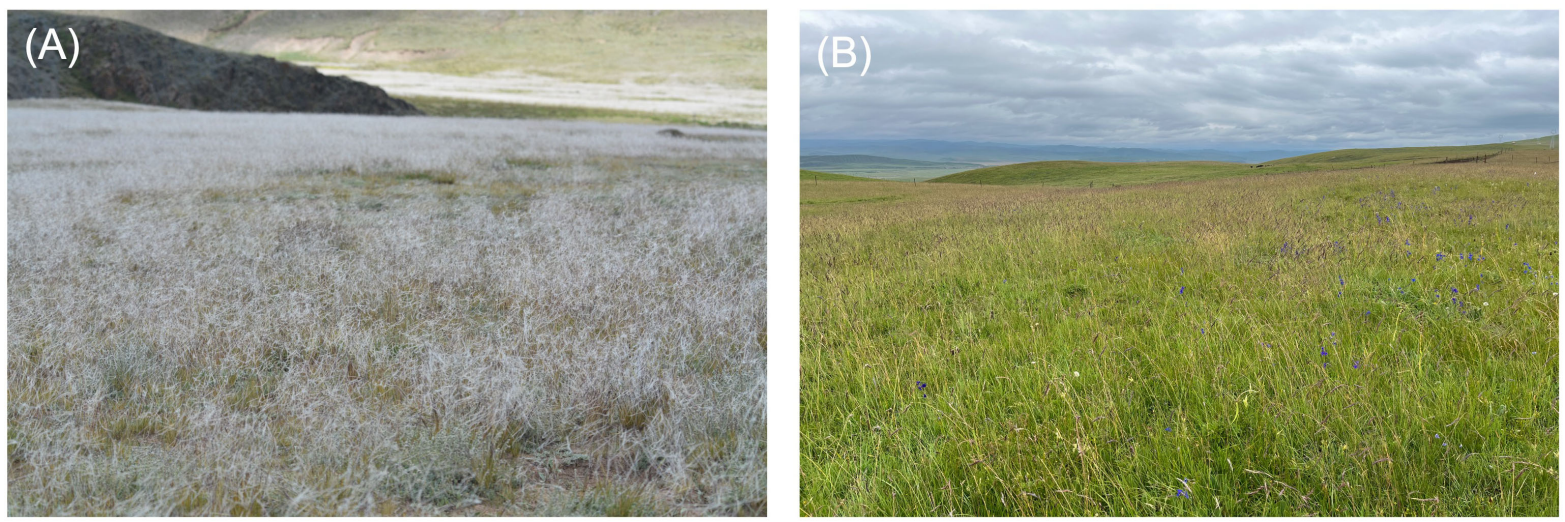
Two main undegraded grassland types of alpine steppe dominated by *Stipa* spp. **(A)** and alpine meadow dominated by tall grasses with *Kobresia* spp. underneath **(B)** on the Tibetan plateau. Alpine grasslands offer numerous material and non-material benefits to humans. These include a variety of ecosystem services such as water supply and regulation, air quality improvement, carbon sequestration, erosion control, and various cultural services, among others. Photos by YN.

## Current state and the causes of Tibetan grassland degradation

Grassland degradation is widespread in many low- to middle-income countries and regions, threatening the livelihoods of hundreds of millions of people, as well as the wildlife and livestock they rely on. One notable hotspot of severe vegetation and soil degradation (Hua et al., 2023) is the Tibetan plateau (Bardgett et al., 2021), the largest grassland area on the Eurasian continent. This region includes the Three-River Headwater area, which supplies water to over a billion people. The causes of Tibetan grassland degradation, whether due to anthropogenic disturbances or climate change (Wu et al., 2021), are still under debate, hindering our understanding of the current drivers and the attribution of degradation. For example, Qiqige et al. demonstrated how climate, not grazing, affects soil microbial diversity through changes in vegetation and abiotic factors across the Eurasian steppe. Over the past four decades, the large increase in livestock numbers has been considered by many scientists to be the major cause of alpine grassland degradation on the Tibetan plateau, which has led to massive degradation and soil erosion of the alpine ecosystems (Niu et al., 2019), regardless of the livestock species, grazing regime, or grassland type. Stocking rates on these alpine grasslands have significantly increased in recent years, with overstocking rates in the range of 27–529% in various regions (Niu et al., 2019). The plateau’s annual temperature rose by 0.34–0.44°C per decade, nearly twice the global average increase of 0.19°C per decade (Meng et al., 2023). Coupled with this significant regional warming, the Tibetan grasslands have faced greater impacts. Additionally, other natural and anthropogenic disturbances including road construction such as the Qinghai-Tibet Highway (Tan et al.), mining (Yang et al.), earthquakes (Zuo et al.), and rodent outbreak (Xu et al.) have also significant effects on plant species distribution, diversity and community structure on the grasslands. So far, we do not have a complete and reliable picture of Tibetan grassland degradation. Xu et al. employed the TSMK-FVC method using Landsat imagery to assess the effects of terrain and climate on vegetation, facilitating large-scale degradation investigations. The estimates of degradation vary for the Tibetan grasslands. The most frequently quoted value is between 30-50% of grasslands on the Tibetan plateau are degraded (Liu et al., 2018; Miehe et al., 2019) with impaired ability to conserve biodiversity and deliver ecosystem services including primary production and key functions such as regulation of hydrology, soil carbon storage and efficient nutrient cycling. For examples, Wu et al. found the significant changes in plant community dynamics and dominant species along the alpine wetland degradation of Qinghai Lake.

## Restoring Tibetan grasslands

Halting and reversing Tibetan grassland degradation is a major challenge in meeting the targets of the UN Decade on Ecosystem Restoration and the 30 by 30 restoration goals of the Kunming-Montreal Global Biodiversity Framework. The restoration must lie first by designing research projects that diagnose the ecological problem and degree of degradation and the key turning points (Li et al.) for different ecosystems, then link the identified problem to the underlying processes or critical components that have been affected (Palmer and Stewart, 2020), finally focus on what can be done to improve restoration outcomes. Wang et al. evaluated the effects of various current restoration measures on short-term grassland restoration. Chen et al. found that improved grazing management can restore *Chnatherum inebrians*-type degraded grassland. Central to scientific advances is the recognition that restoration efforts must focus on re-establishing ecological processes and the strong interactions among different trophic levels. Li et al. also shown the key role of microbes in restoring alpine desertified grasslands. However, Tibetan restoration efforts are at risk of failing without fundamental knowledge about the reproductive capacity of plant species during degradation, especially the clonal propagation in this cold region. Li et al. shown the correlation between bud diversity and community composition in different degraded alpine grasslands. Furthermore, the restoration process should not focus solely on improving a single function, such as production (Wang et al.), but on enhancing multifunctionality, including biodiversity. Shu et al. demonstrated that mixed and diverse shrub-grass planting enhances both biodiversity and ecosystem stability.

Facing the strong need for promoting ecosystem restoration that sustainably achieves socio-economic-natural benefits on Tibetan plateau, scientific research plays a vital role in developing and refining dynamic restoration strategies that are efficient, cost-effective, suitable for such sensitive ecosystems, ensuring the long-term sustainability and resilience of restored areas. Importantly, nature conservation and preventing Tibetan grassland degradation should have priority over restoration.

## Author contributions

YN: Conceptualization, Investigation, Methodology, Supervision, Validation, Writing – original draft, Writing – review & editing. YB: Conceptualization, Funding acquisition, Investigation, Methodology, Validation, Writing – review & editing. SR: Conceptualization, Investigation, Methodology, Validation, Writing – review & editing.
